# Advancing HIV and Cancer Research: Implementing an AIDS and Cancer Specimen Resource in Latin America

**DOI:** 10.1177/19475535251363861

**Published:** 2025-08-08

**Authors:** Larissa L.S. Scholte, Evandro S. Mello, Karim Yaqub Ibrahim, Miyuki Uno, Vanesse Maria da Costa, Ellen Sayuri Miazima, Camila Motta Venchiarutti Moniz, Giullia Dias de Souza, Larissa Oliveira Amorim, Isabela Cristina de Souza, Kris Oliveira, Roger Chammas, Kristina Bowles, Lipsa Das, Anna E. Coghill, Lisa Rimsza, Aluisio Segurado, Jeffrey M. Bethony

**Affiliations:** ^1^ Department of Microbiology, Immunology and Tropical Medicine, The George Washington University, Washington, District of Columbia, USA.; ^2^ Faculdade de Medicina, Universidade de São Paulo, São Paulo, Brazil.; ^3^ Center for Translational Research in Oncology, Instituto do Câncer do Estado de São Paulo, Hospital das Clinicas da Faculdade de Medicina da Universidade de São Paulo, São Paulo, Brazil.; ^4^ Comprehensive Center for Precision Oncology, Universidade de São Paulo, São Paulo, Brazil.; ^5^ Center for Immunization and Infection Research in Cancer, Cancer Epidemiology Program, Moffitt Cancer Center, Tampa, Florida, USA.; ^6^ Department of Pathology, University of Arizona, Tucson, Arizona, USA.

**Keywords:** HIV, cancer, biorepository, translational research

## Abstract

The AIDS and Cancer Specimen Resource (ACSR) has developed a global biorepository network to support research on AIDS-defining and non-AIDS-defining cancers. This article details the establishment of a dedicated HIV-associated cancer biorepository in São Paulo, Brazil, a region with a high burden of these malignancies. The repository addresses the need for high-quality, well-annotated biospecimens from Latin American (LATAM) populations to support research on cancer pathogenesis in people with HIV (PWH), viral reservoirs, and clinical outcomes. It systematically collects and links biospecimens with demographic and clinical data, providing a resource for investigators. Developed with international ethics, community engagement, and regulatory standards, the biorepository is modeled after similar efforts in low- and middle-income countries. This article outlines its implementation, including sample acquisition, infrastructure, inventory management, data governance, and research collaboration. By expanding access to biospecimens, the ACSR supports research that can improve outcomes for PWH and cancer, while strengthening research capacity in the LATAM region.

## Origin, Evolution, and Structure of the AIDS and Cancer Specimen Resource

**FIG. 1.**
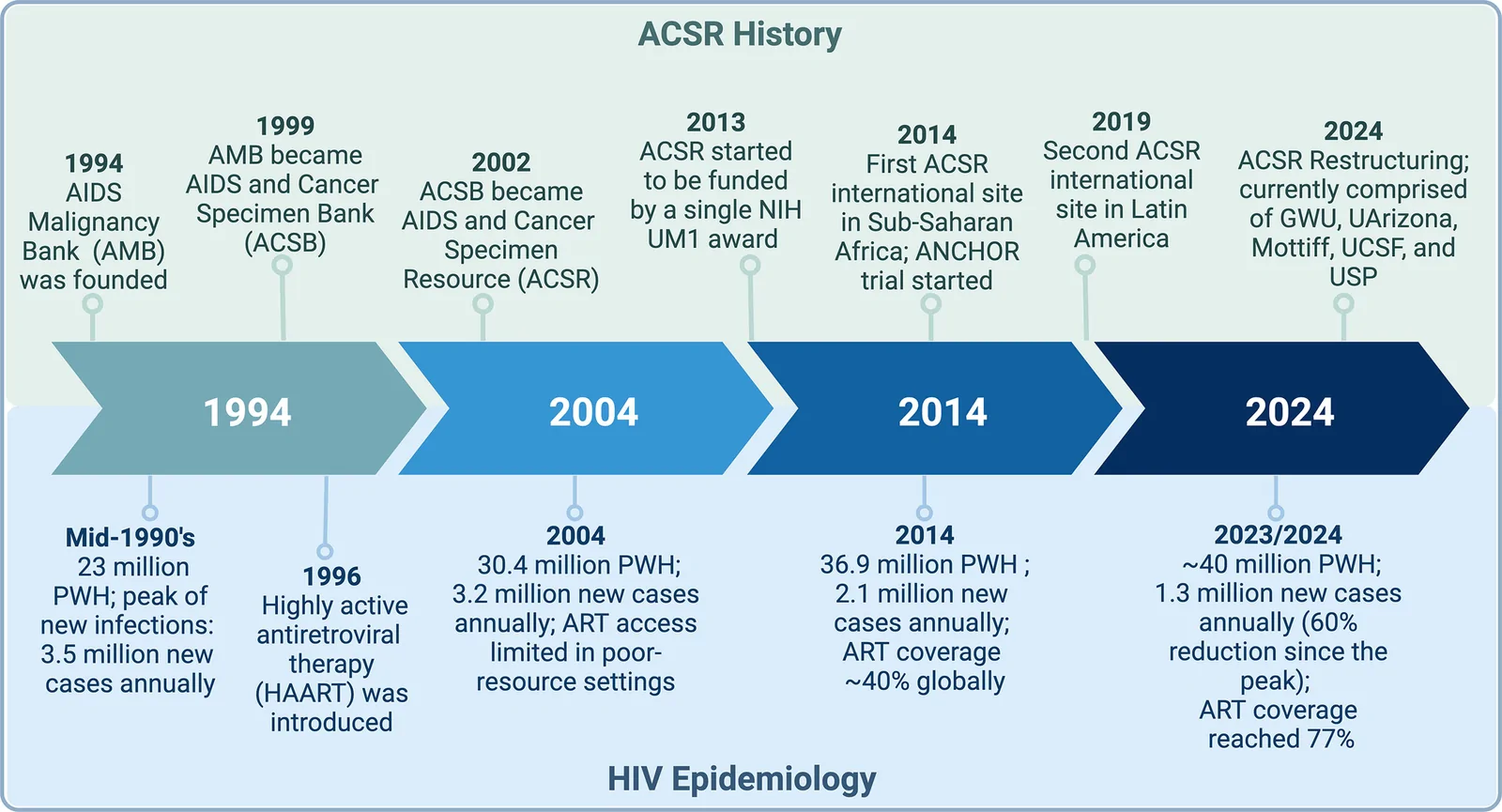
AIDS and Cancer Specimen Resource history.^1^

The AIDS and Cancer Specimen Resource (ACSR) was established in 1994 and is funded by the National Cancer Institute (NCI), a division of the National Institutes of Health (NIH) in the United States. The ACSR supports basic and translational research on HIV-associated malignancies by providing researchers with access to well-characterized biospecimens and clinical data. Over the past three decades, the ACSR has evolved from independent biobanks into a unified consortium funded through a UM1 cooperative agreement with the NCI. This transition has streamlined operations, improved data consistency, enhanced cost-effectiveness, fostered interdisciplinary collaboration, and accelerated scientific discoveries (Fig. 1).^1^

ACSR specimens are provided at no cost and include a diverse collection of sample types from individuals with and without HIV infection. The repository contains specimens from AIDS-defining cancers (ADCs), including Kaposi sarcoma, non-Hodgkin lymphoma—particularly aggressive B cell lymphoma—and non-AIDS-defining cancers (NADCs), such as breast and lung (nonviral, age-related etiology), and anal (virus-associated) cancers. Critically, these samples span both the pre- and post-antiretroviral therapy (ART) eras, offering valuable insight into the evolving landscape of HIV-associated cancers over time.

**FIG. 2.**
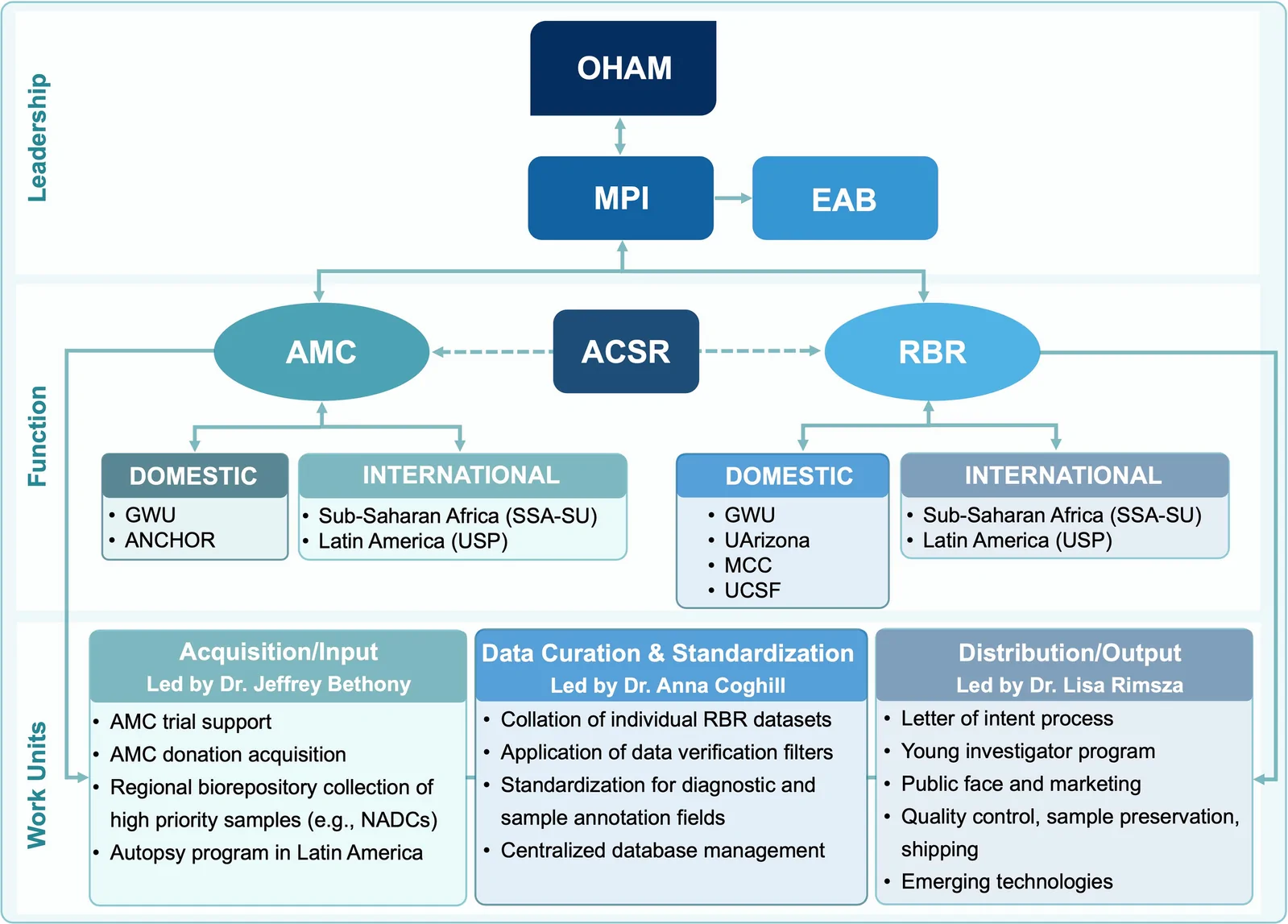
ACSR organizational structure. New structure of ACSR includes a multidisciplinary MPI team to which all six regional biorepositories report directly. These RBRs have both AMC clinical trials and ACSR (nonclinical trials) functions. Input comes to the MPI team from OHAM and an EAB. ACSR, AIDS and Cancer Specimen Resource; OHAM, Office of HIV and AIDS Malignancy; MPI, multiple principal investigators; EAB, External Advisory Committee; AMC, AIDS Malignancy Consortium; RBR, Regional Biospecimen Repository; GWU, George Washington University; ANCHOR, Anal Cancer HSIL Outcomes Research; SSA, Sub-Saharan Africa; SU, Stellenbosch University; USP, University of São Paulo; UArizona, University of Arizona; UCSF, University of California San Francisco; MCC, Moffitt Cancer Center.

The ACSR’s current operating structure (Fig. 2) includes six Regional Biospecimen Repositories (RBRs), four of which also serve as AIDS Malignancy Consortium (AMC) Biorepository Units. These RBRs support two key aims^1^: maintaining biorepository services for AMC clinical trials in accordance with best practices from the College of American Pathologists (CAP), the NCI, and the International Society for Biological and Environmental Repositories (ISBER)^2^; and RBR operations including specimen acquisition, data management, and distribution to support HIV and cancer research. The RBRs are located at George Washington University (GWU), the University of Arizona (UA), Moffitt Cancer Center (MCC), the University of California San Francisco (UCSF), Stellenbosch University (SU, South Africa), and the University of São Paulo (USP, Brazil), selected for their capacity to acquire HIV and HIV-associated malignancy specimens.

### Advancing HIV-related cancer science through the AIDS and Cancer Specimen Resource biobank

The ACSR follows a biobanking model that emphasizes standardized collection, processing, and long-term storage of biospecimens spanning both pre- and post-ART eras.^1^ The ACSR is distinguished by its informatics infrastructure and expert clinical annotation that comprehensively link HIV status, related comorbidities, and cancer diagnoses. This integration, requiring deep expertise in both HIV and oncology, makes the ACSR a uniquely valuable resource for advancing research in HIV-associated malignancies. This integration requires specialized expertise in both HIV and oncology, establishing the ACSR as an unparalleled resource in the field. The ACSR supports the AMC through a network of domestic and international biorepositories operating under the umbrella of the AMC Biorepository Program, which the ACSR at GWU oversees. The program provides critical infrastructure for AMC clinical trials by processing, managing the inventory, and distributing biospecimens collected during trials to the appropriate Network Resource Laboratories within the consortium. Several key studies are listed below.

Currently, the AMC Domestic Biorepository at GWU manages approximately 50,000 specimens, which include plasma, serum, whole blood, and tissue collected from joint AIDS Clinical Trial Group and AMC studies conducted in Sub-Saharan Africa (SSA). These trials evaluated: (1) the efficacy of immediate versus delayed etoposide in combination with ART for limited-stage Kaposi’s sarcoma^2^; and (2) the WT1 expression in Kaposi sarcoma tumors from HIV-associated and non-HIV cases, examined Kaposi Sarcoma Herpesvirus (KSHV)’s role in regulating WT1, and explored the effects of WT1 suppression during latent KSHV infection *in vitro*.^3^ These efforts have advanced understanding of Kaposi sarcoma-associated herpesvirus pathogenesis, as well as the safety and toxicity profiles of combination therapy. Other notable findings supported by ACSR specimens include the identification of mast cells as pro-inflammatory reservoirs activated by KSHV within Kaposi sarcoma lesions and a dose-dependent relationship between plasma immunoglobulin E levels and Kaposi sarcoma severity, implicating type 2 inflammation in disease progression.^4,5^ The use of tissue microarrays (TMAs) has enabled high-throughput molecular analyses, facilitating the discovery of viral circular RNAs derived from Epstein–Barr virus and KSHV in tumor tissues, which may serve as novel biomarkers.^6^ Furthermore, molecular characterization of germinal center B cell diffuse large B cell lymphoma in people with HIV (PWH) has revealed distinct transcriptional, genomic, and protein expression profiles compared with HIV-negative cases, including reduced BCL2 expression, suggesting altered apoptotic regulation in the HIV-positive (HIV+) setting.

For a broader overview of the ACSR’s scientific impact, see Silver et al.^1^ Collectively, these contributions highlight the crucial role of the ACSR’s integrated biobanking and informatics platform in driving translational research, enhancing mechanistic understanding, and informing therapeutic approaches for HIV-associated malignancies worldwide.

### Integration of AMC and ACSR biorepository infrastructure

The AMC, established in 1995, is an NCI-supported clinical trials group dedicated to investigating new treatment and preventive solutions interventions for malignancies in PWH and studying the pathobiology of these tumors within the context of clinical trials. The AMC operates globally, comprising over 36 Clinical Trial Sites across the United States, SSA, and Latin America (LATAM), organized into five specialized working groups: Kaposi Sarcoma, Hematological Malignancies, Human Papillomavirus (HPV)-associated Cancers, Solid Tumors, and a Laboratory Resources Committee (amcoperations.com).

The AMC Biorepository Program’s infrastructure is interconnected with the rest of the ACSR, as it shares physical infrastructure, staff, and standardized operating procedures (SOPs) with the ACSR RBRs. This ensures personnel availability, adherence to the ACSR Quality Management Plan (QMP), compliance with best practices, and accurate sample annotation in inventory control systems, such as Freezerworks (Dataworks Development Inc., Mountlake Terrace, Washington, USA) and ACSR Tissue Locator and Annotation System (ATLAS). When specific AMC protocols require deviations, they are explicitly detailed in each study’s Biorepository Manual of Procedures.

The AMC SSA Biorepository Unit at SU exemplifies this integrated model, sharing infrastructure and procedures with the SSA ACSR RBR. This unit not only ensures standardized sample processing and storage but also reviews AMC protocols before study initiation, recommending changes based on best practices when necessary.

The AMC is expanding its international presence through the development of a clinical trial network in Brazil, Argentina, and Mexico. This initiative aims to conduct multinational clinical trials specifically to treat and prevent cancers in PWH, improve research infrastructure-including clinic facilities and laboratories-, data management, specimen acquisition, storage capabilities, facilitate training and retention of cancer-trained investigators, pathologists, and study staff in these countries.

The AMC Biorepository at the UA is the only protocol-specific biorepository, as it stores samples from the AMC-supported Anal Cancer for high-grade squamous intraepithelial lesions (HSIL) Outcomes Research Study (ANCHOR). This large, NCI-funded, multicenter clinical trial was designed to determine whether treating Anal HSIL in the anus can prevent progression to anal cancer in PWH, paralleling the cervical HSIL treatment approach used to prevent cervical cancer.^7,8^ The study screened 10,723 PWH for anal HSIL, and 4459 participants with biopsy-confirmed HSIL were randomized to either active monitoring or treatment using ablative therapies or topical drugs. Participants were followed every 6 months with digital anorectal examinations, anal cytology, and high-resolution anoscopy, and all consented to the use of their biospecimens in protocol-defined research related to anal HSIL, anal cancer, and HPV.^8^ The ANCHOR Biorepository receives specimens from participating sites and coordinates specimen preparation and shipment to approved research laboratories, including academic, governmental, and commercial partners. Residual materials from distributed specimens are returned, re-inventoried, and stored. The current ANCHOR inventory includes samples from 10,885 screened participants, 4535 randomized participants, and 2990 post-enrolled participants.

### Contextualizing the need for AIDS and cancer biorepositories in Latin America

Ensuring sample quality in resource-limited settings is challenging, underscoring the need for collaboratively managed repositories. At the USP, a model integrating clinical teams, the pathology department, and biorepository staff has been successfully implemented to support research aimed at improving outcomes for PWH and cancer. According to UNAIDS, out of 39.9 million, 30.7 million PWH worldwide received ART in 2023 (∼79% of all PWH), with 28.6 million (93.1%) achieving viral suppression (i.e., undetectable plasma HIV loads). Among them, 2.3 million live in LATAM.^9^ Brazil stands out in this region with the most significant number of PWH (1 million), of whom 786,893 received ART, with 75% achieving viral suppression.^9^ Nevertheless, despite considerable progress in expanding access to ART and HIV pre-exposure prophylaxis (PrEP), HIV incidence rates have remained persistently high in LATAM countries. The epidemic remains concentrated in large urban centers and disproportionately impacts vulnerable populations, with men who have sex with men (MSM) and transgender women suffering the highest burden. Regional data indicate that HIV prevalence surpasses 10% in these populations, with no significant decline in new infections over the past 15 years.^10^ In this context, Brazil continues to face the burden of HIV infection and AIDS, particularly among its vulnerable population groups—MSM, sex workers, transgender people, and people who suffer from substance use disorders.

The “test and treat strategy,” which advocates for ART for all PWH regardless of immune status, and PrEP, were implemented in Brazil with universal access through the public Unified Health System in 2013 and 2018. These measures have significantly reduced AIDS detection rates (from 21.9 per 100,000 inhabitants in 2012 to 17.8 per 100,000 inhabitants in 2023). Additionally, AIDS mortality remains a concern, with 10,338 reported AIDS-related deaths in 2023, corresponding to a standardized mortality rate of 3.9 per 100,000 inhabitants.^11^ In terms of disease distribution, there is a significant concentration of AIDS cases in the densely populated Southeast region, where São Paulo, Brazil’s most populous state, is located, accounting for 51% of all reported cases. The most affected age groups are young people aged 15–24 years (23.2%) and adults aged 25–34 years (34.9%).^11^

**FIG. 3.**
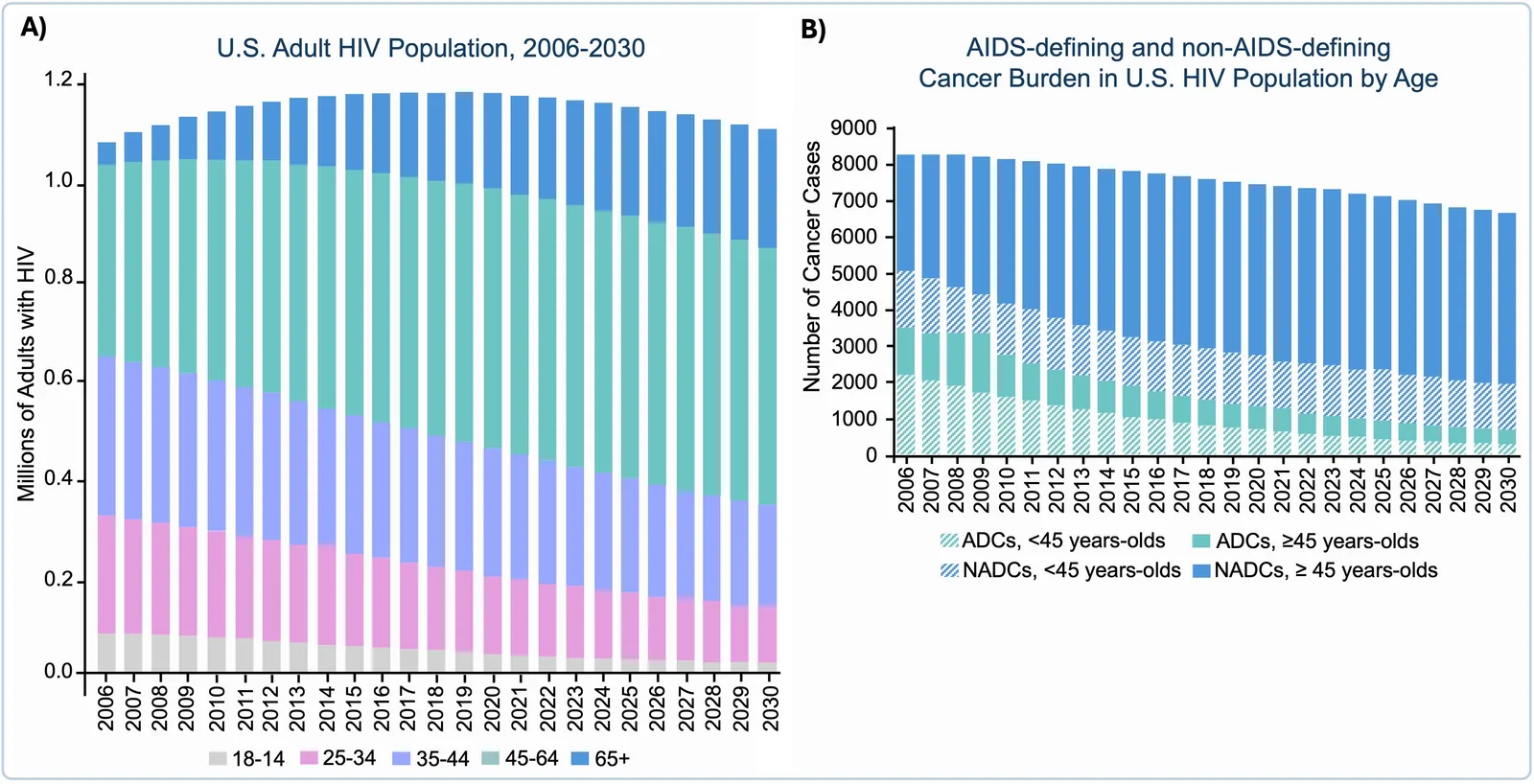
**(A)** Estimated adult population living with HIV in the United States during the last two decades. **(B)** Estimated cases of ADCs and NADCs in the adult HIV+ population. Modified from Shiels et al.^12^ ADC, AIDS-defining cancer; NADC, non-AIDS-defining cancer.

In recent years, due to the increased survival among PWH, the management of comorbidities—including cardiovascular disease and malignancies—has become a matter of public health concern. The success of ART has significantly increased the life expectancy of PWH in both resource-rich and resource-limited areas of the world, leading to an aging HIV+ population (Fig. 3A).^12,13^ Consequently, the proportion of PWH over 60 years of age in the United States has grown, shifting the primary health concern to NADCs, such as lung and prostate cancer, which are becoming more prevalent than ADCs (Fig. 3B).^12^ As the PWH population ages, continued interdisciplinary research will be crucial to ensure long-term health and quality of life, and we expect this aging trend among PWH to also be present in LATAM with increased access to effective HIV therapy.

Notably, rising trends in cancer-related mortality within this population have been reported in Brazil.^14^ In metropolitan São Paulo, the proportion of cancer-related deaths in PWH increased from 6.2% before the introduction of combined ART (1991–1996) to 10.1% in the combined ART era (2000–2006), a trend likely to continue as this population lives longer.^15^

The Instituto do Câncer do Estado de São Paulo (ICESP) serves as a reference center for HIV-associated malignancies. It receives patients with cancer referred from the busiest HIV clinics in the metropolitan São Paulo region, including the Centro de Referência e Treinamento Sexually Transmitted Infections/-AIDS (an HIV reference center affiliated with the São Paulo State Department of Health), the Instituto de Infectologia Emílio Ribas (an Infectious Diseases Reference Hospital), as well as from other cities within the state of São Paulo. Notably, approximately 20% of the patients admitted to ICESP are referred by the Academic Health Center of Hospital das Clínicas da Faculdade de Medicina da Universidade de São Paulo, having initially been followed at the University-affiliated HIV Clinic (SEAP). Between 2009 and 2019, ICESP admitted and treated 917 patients with HIV-associated malignancies, 73% of whom had NADCs—53% non-virus-related and 20% virus-related—while 27% had ADCs. The most common malignancies, including ADC and NADC, were non-Hodgkin lymphoma (14.7%), anal cancer (9.9%), and Hodgkin lymphoma (6.5%). Other cancers included Kaposi sarcoma, breast cancer, liver and biliary tract cancers, as well as uterine, prostate, and lung cancers. During this period, 298 of the 917 patients (32.5%) in the cohort died. Given the significant burden of HIV-associated cancers in São Paulo—one of the most ethnically diverse regions in the world—and the heterogeneous clinical profiles of affected patients, establishing a dedicated AIDS/HIV biorepository in this region is essential to advancing research aimed at improving health outcomes and guiding evidence-based care for PWH.

While the current operational hub is based in São Paulo (ICESP), the long-term vision for the ACSR includes expanding biospecimen collection and data harmonization efforts across Brazil and other Latin American countries. These collaborations aim to broaden the geographic and demographic catchment area and enhance sample acquisition, particularly from underrepresented regions. Expansion to additional institutions will follow standardized protocols for specimen handling, data sharing, and ethical oversight to ensure quality and harmonization across all sites, guided by feasibility assessments and regulatory preparedness.

### Establishment of an AIDS and Cancer Specimen Resource in Brazil

#### Regulatory, oversight, and logistics of operations in Brazil

The ACSR in Brazil was established as a dynamic, ongoing biobank aimed at supporting translational research on HIV-associated malignancies within the regional context. Rather than setting a fixed target for specimen accrual, this resource prioritizes the systematic collection and curation of high-quality, clinically annotated biospecimens that reflect the evolving epidemiology and treatment landscape of HIV and associated cancers in Brazil. The biobank’s inventory will include a diverse range of specimen types, such as tissue biopsies, plasma, serum, and whole blood collected from cohorts during this post-ART era. This breadth ensures that the resource captures relevant clinical heterogeneity, facilitating longitudinal and comparative studies. Success for the Brazilian ACSR is defined by its ability to maintain and expand a well-annotated collection that meets the research needs of the scientific community. This is supported by continuous evaluation of specimen utilization, quality control metrics, and investigator demand, allowing the biobank to adapt its collection strategies responsively. Integral to this effort is the incorporation of robust informatics systems that link biospecimens to detailed clinical and demographic data, enhancing the utility of the collection for biomarker discovery and precision oncology. By focusing on both traditional biospecimen management and advanced data integration, the Brazilian ACSR serves as a critical infrastructure for advancing understanding, diagnosis, and treatment of HIV-associated cancers in Brazil and beyond.

Implementing a biorepository in Brazil required strict adherence to both national and international regulatory standards, which are essential for ensuring data security and safeguarding participant privacy. The country maintains a well-established system for ethical oversight of human research, coordinated by the National Health Council (CNS) and composed of the National Ethics C ommittee for Research (CONEP) and a network of institutional Research Ethics Committees (CEPs) distributed nationwide.^16^ Importantly, Brazil makes a regulatory distinction between biorepositories and biobanks, which differs from definitions in many other countries. Brazilian legislation classifies biorepositories as collections of human biological materials intended for use in specific, predefined research projects, with a limited scope and duration. In contrast, biobanks are established for indefinite storage and future use of biospecimens in multiple research studies, requiring additional ethical approvals for each new project involving the use of archival biospecimens. This distinction has significant implications for governance, ethical review processes, and compliance requirements, and reflects Brazil’s commitment to ensuring participant autonomy, privacy, and informed consent in human subject research.

In this context, establishing the LATAM ACSR at the USP demanded transparent governance, including clearly defined decision-making processes, accountability structures, and oversight committees to ensure compliance with ethical and regulatory guidelines. Key regulatory documents include the CNS Resolution no. 441/2011^17^ and the Ministry of Health Ordinance no. 2201/2011,^18^ which provide standards for implementing biorepositories and biobanks. Additional compliance was ensured with regulations governing research involving human subjects, access to information, and general data protection, including CNS Resolution no. 466/2012,^19^ Law no. 14.874/2024,^20^ Law no. 12.527/2011,^21^ and Law no. 13.853/2019^22^ (Table 1). These regulatory documents undergo periodic revisions to remain aligned with evolving research practices, data protection protocols, and international ethical standards. The ACSR LATAM research project was developed in compliance with the Brazilian legislation, reviewed and approved by both the local CEP and the CONEP. Ethical approval was granted through Letter of Approval no. 3.236.441, issued on June 4, 2019, and subsequently amended under approval no. 6.989.931, dated December 8, 2024. The LATAM biorepository functions as a sample subcollection within the Academic Biobank for Cancer Research Network at USP. It is officially designated as an RBR under amendment CONEP Approval no. 16/2022.

**Table 1.** Main Brazilian Regulatory Documents Related to Biobanks, Human Research, and Data Protection

Main area	Regulatory documents	Reference
Biorepositories and biobanks	CNS Resolution no. 441/2011	^17^
	Ministry of Health Ordinance no. 2201/2011	^18^
Research involving human subjects	CNS Resolution no. 466/2012	^19^
	Law no. 14.874/2024	^20^
Access to information, and general data protection	Law no. 12.527/2011	^21^
	Law no. 13.853/2019	^22^

CNS, “*Conselho Nacional da Saúde*” (in Portuguese)—National Health Council.

Complementing these legal and ethical requirements, regulatory oversight by the National Health Surveillance Agency in Brazil (ANVISA)^23^ plays a critical role in ensuring the operational integrity of biorepository and biobanking facilities, particularly when clinical research is involved. ANVISA operates across the entire national territory, with a primary mandate to safeguard public health through the sanitary regulation of the processes related to the production, distribution, and consumption of products and services. It also oversees all activities related to sanitary surveillance, including establishing and enforcing technical regulations for health services and managing waste generated by biorepositories and biobanks.

In parallel, the shipping of biological materials requires thorough consideration of the type of material, strict adherence to regulatory requirements, proper packaging assembly, accurate labeling, and the selection of reputable carriers. In Brazil, such shipments must comply with the Collegiate Directorate Resolution no. 20/2014,^24^ issued by ANVISA, which regulates the transportation of biological material from a health surveillance perspective. This resolution outlines the standards for shipping services engaged in the transport of biological materials on behalf of entities such as clinical laboratories, hospitals, clinics, cell banks, tissue banks, and similar institutions, using either in-house or third-party logistics infrastructure.^25^ Moreover, compliance with regulations issued by additional transportation authorities is mandatory. These include the National Civil Aviation Agency (ANAC),^26^ the National Land Transportation Agency (ANTT), and the National Traffic Department (CONTRAN), among others.

#### Progress and inventory overview of the LATAM ACSR

**FIG. 4.**
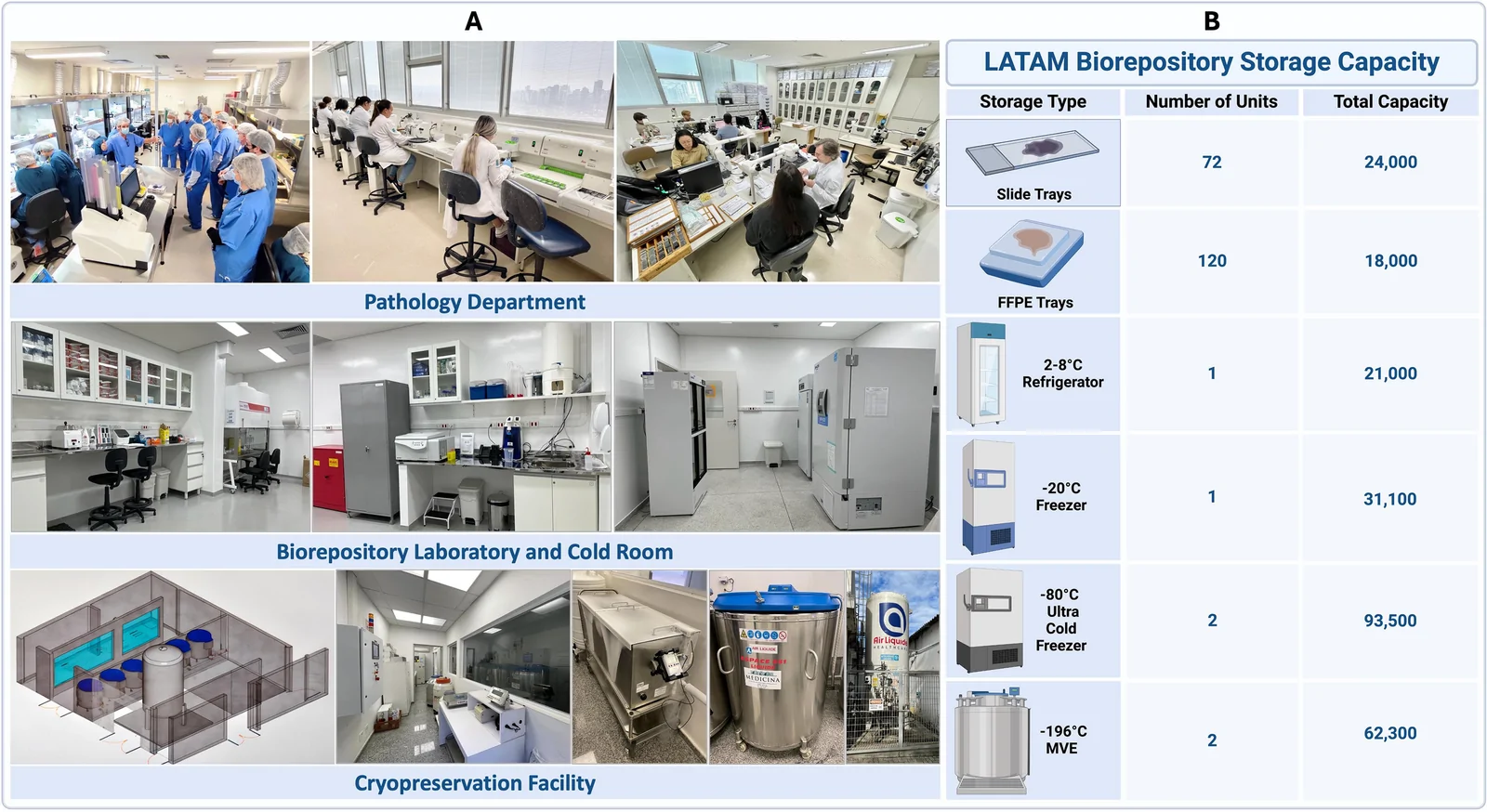
**(A)** LATAM ACSR biorepository infrastructure. Sample collection and curation are performed by the biorepository team and pathology department staff at ICESP. The biorepository is composed of a laboratory, a cold room, a cryopreservation facility, and an office and storage room (not shown). **(B)** Total biorepository storage capacity. LATAM, Latin American; ACSR, AIDS and Cancer Specimen Resource; ICESP, Instituto do Câncer do Estado de São Paulo.

**FIG. 5.**
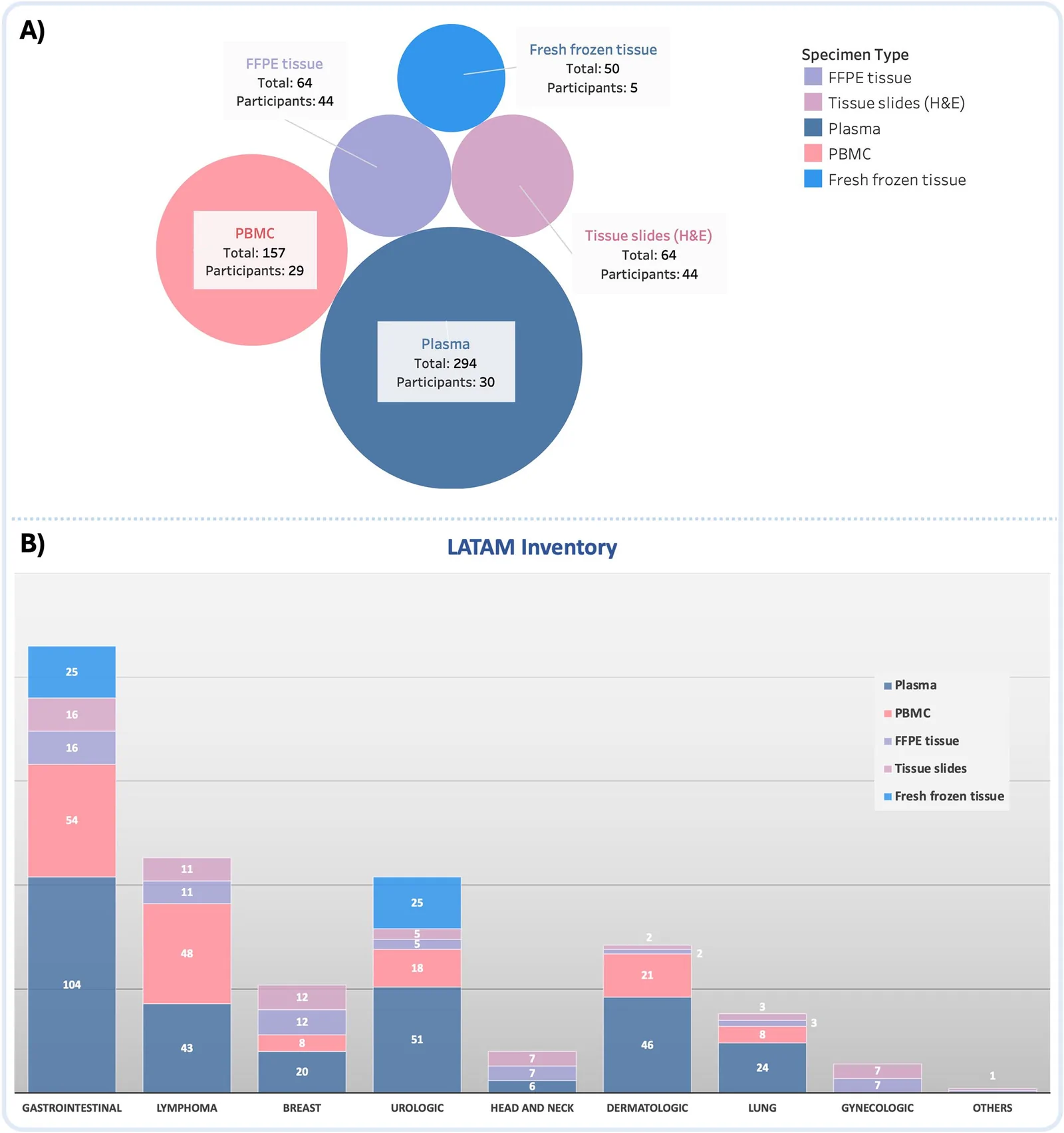
Aliquot-level inventory of samples that have been processed and/or stored at the LATAM ACSR RBR at the University of São Paulo, as of January 31, 2025. **(A)** Six hundred twenty-nine specimens collected from 73 participants categorized per sample type. *Circle size* indicates the number of specimens stored relative to others. **(B)** Number of specimens per specimen type and tumor specialty. LATAM, Latin American; ACSR, AIDS and Cancer Specimen Resource; RBR, Regional Biospecimen Repository.

The success of the ACSR in Brazil is not measured solely by the number of samples collected, although the sample accrual has been robust, particularly compared with other international ACSR sites. Instead, true success is determined by the utilization of these biospecimens by the research community. The Latin American ACSR biorepository, operational at full capacity since December 2023, has already provided samples from PWH and associated malignancies to investigators, and several publications are anticipated. However, given the recent activation of these collections and their initial use, it is premature to fully assess the impact of this resource. To date, the LATAM inventory comprises biospecimens from 73 donors, all receiving ART, with 86.5% of those tested showing undetectable viral loads. Most participants (86.7%) were diagnosed with NADCs, predominantly gastrointestinal malignancies, followed by breast and urologic cancers, while nine donors had ADCs, including lymphomas, Kaposi sarcomas, and cervical cance rs (Fig. 4). Key investments in infrastructure, including modern storage facilities, backup power, and environmental controls, have been implemented to ensure specimen integrity and long-term sustainability. Ongoing efforts focus on optimizing sample quality and annotation to maximize research value. Figure 5 and Supplementary Table S1 detail the current LATAM ACSR aliquot-level inventory by sample type and tumor specialty, underscoring the diversity and clinical relevance of the collection.

### Strategies in place for improving sample collection and biorepository sustainability

#### Participant identification

**FIG. 6.**
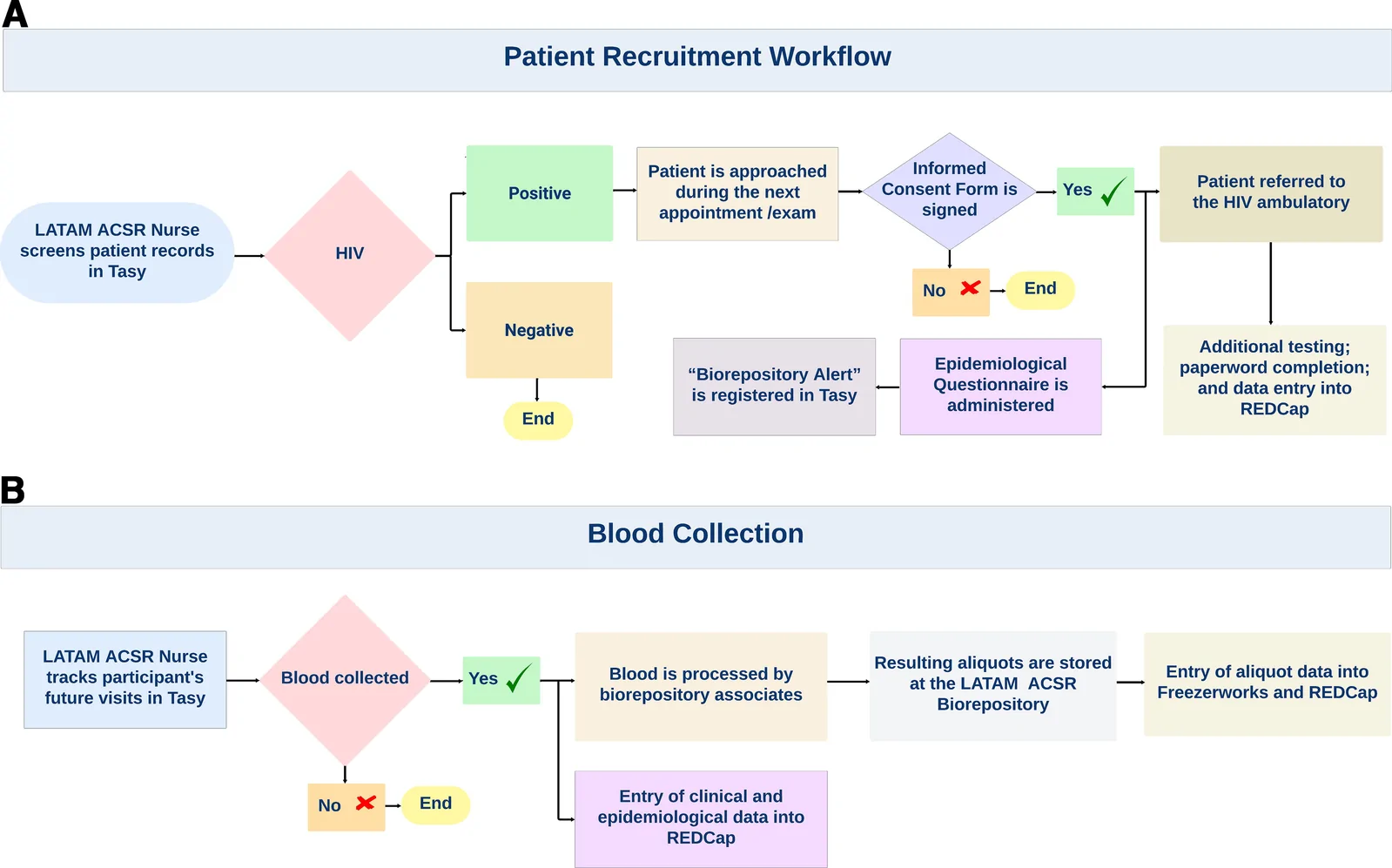
Participant enrollment and biospecimen workflow. **(A)** Patient recruitment workflow. **(B)** Blood sample collection, processing, storage, and data management workflow.

The LATAM ACSR employs a structured and integrated approach to identify and enroll PWH and HIV-associated cancers. At ICESP, participant tracking and recruitment are facilitated through the Tasy Delphi electronic medical record system (Philips Medical Systems Nederland B.V.). The enrollment process is led by a dedicated LATAM ACSR nurse, who reviews appointment schedules to identify potential participants meeting the study’s inclusion criteria. Eligible patients are approached during their routine visits. Upon agreement to participate, the Informed Consent Form is signed, and the Epidemiological Questionnaire is completed. Following consent, a baseline blood sample is collected, processed, and stored by trained LATAM Biorepository Associates. Participants are then referred to the infectious diseases outpatient clinic, and a “Biorepository Flag” is entered into the electronic system to facilitate tracking of future visits and coordination of subsequent sample collections. The patient recruitment and sample management workflows are summarized in Figure 6.

#### Recruitment and sample collection

Once identified, eligible participants are approached by the ACSR nurse during routine appointments. Upon consent, clinical and epidemiological data are entered into Research Electronic Data Capture (REDCap), and patients are flagged in Tasy Delphi for future tracking. Blood samples are collected during scheduled visits, processed into research aliquots, and prepared for long-term storage with accompanying annotation in both REDCap and Freezerworks.

#### Tissue collection

For participants undergoing surgery, coordination with surgical and pathology teams ensures timely collection of tumors and matched non-lesional tissues. The pathologist prioritizes diagnostic needs but also selects research-appropriate specimens. Tumor and non-tumor samples are snap-frozen in liquid nitrogen (i.e., for future DNA, RNA, and protein analysis) and logged with cold ischemia and fixation times to ensure integrity. Hematoxylin and eosin-stained slides confirm tumor content. Storage protocols maintain the quality of formalin-fixed paraffin-embedded tissues.

#### Longitudinal cohort design

The LATAM ACSR emphasizes longitudinal collections from the same individuals at multiple clinical time points, including before, during, and after treatment. This design enables dynamic assessment of disease progression and biomarker changes, supporting studies on HIV-related oncogenesis, treatment response, and immune modulation over time.

#### Unique integration capacity

While the biobanking infrastructure at LATAM ACSR aligns with global standards, its unique strength lies in the integration of HIV and cancer data streams. Successfully navigating distinct medical systems and registries to identify, enroll, and longitudinally track PWH with cancer requires expertise across both domains. This capacity to bridge disparate datasets and build a deeply annotated cohort is what distinguishes the LATAM ACSR within the global biobanking landscape.

### Implementation of an autopsy program

Autopsies represent a critical yet often underutilized opportunity for the collection of high-quality biospecimens in the study of HIV cure and HIV/AIDS-associated malignancies.^27,28^ They offer a rare chance to obtain well-annotated, curated tissue and body fluid samples—including those that are typically inaccessible through routine clinical biopsies. These postmortem collections support basic and translational research, enabling discoveries that would not be possible through standard clinical sampling alone.^29–32^

A medical autopsy involves examining the external body and internal organs of a deceased individual, typically performed by a pathologist to determine or confirm the cause of death. Beyond its primary diagnostic value, the autopsy serves as a powerful tool for research, medical education, and health care quality assurance. One of its most significant advantages is the ability to collect and preserve tissue samples for biobanking and future scientific investigations.

Autopsies offer several key advantages over surgical specimens for biobanking. While surgical collections are limited to specific organs or regions, autopsies allow for the comprehensive retrieval of tissues from multiple systems, providing a more holistic view of disease progression and systemic effects. Furthermore, autopsies also enable the paired collection of pathological and normal tissues from the same individual—an approach not feasible during surgery and essential for comparative studies. Deep-seated anatomical structures, such as the brain, bone marrow, and heart, rarely accessible during surgical procedures, can also be effectively sampled postmortem.

In the context of HIV research, although the virus has b een identified in numerous tissues, most human studies have centered on blood samples, with only limited examination of cerebrospinal fluid or genital secretions. Autopsy-based studies expand this scope, allowing for the collection of a wide range of tissues at the time of death and providing unique insights into the distribution of HIV and the relationship between tissue reservoirs and circulating blood. This is especially important, as peripheral blood primarily contains naïve T cells, which do not fully reflect HIV dynamics in tissues, where differentiated effector memory T cells are more prevalent.

Despite these scientific advantages, the global rate of both clinical and academic autopsies has declined significantly in recent decades. This trend is attributed to a range of factors, including increasing reliance on advanced imaging technologies and clinical laboratory diagnostics, limited communication between health care providers and families, and a general lack of public awareness regarding the continued value of autopsy in improving patient care and advancing science.

Considering these challenges and the transformative potential, the implementation of a LATAM Autopsy program represents a strategic advancement for the ACSR. The initiative is being developed in partnership with the Autopsy Service affiliated with USP within the Hospital das Clínicas Complex. The ICESP will serve as the primary source for potential participants, ensuring a steady and reliable flow of cases. Currently, over 600 PWH and cancer are registered in the ICESP medical system. The biorepository will actively monitor the health care database to identify eligible participants. Infectious diseases clinical staff will flag participants upon admission to intensive care, and biorepository staff will be notified upon the participant's death and body availability to initiate mobilization and preparation for sample collection.

**FIG. 7.**
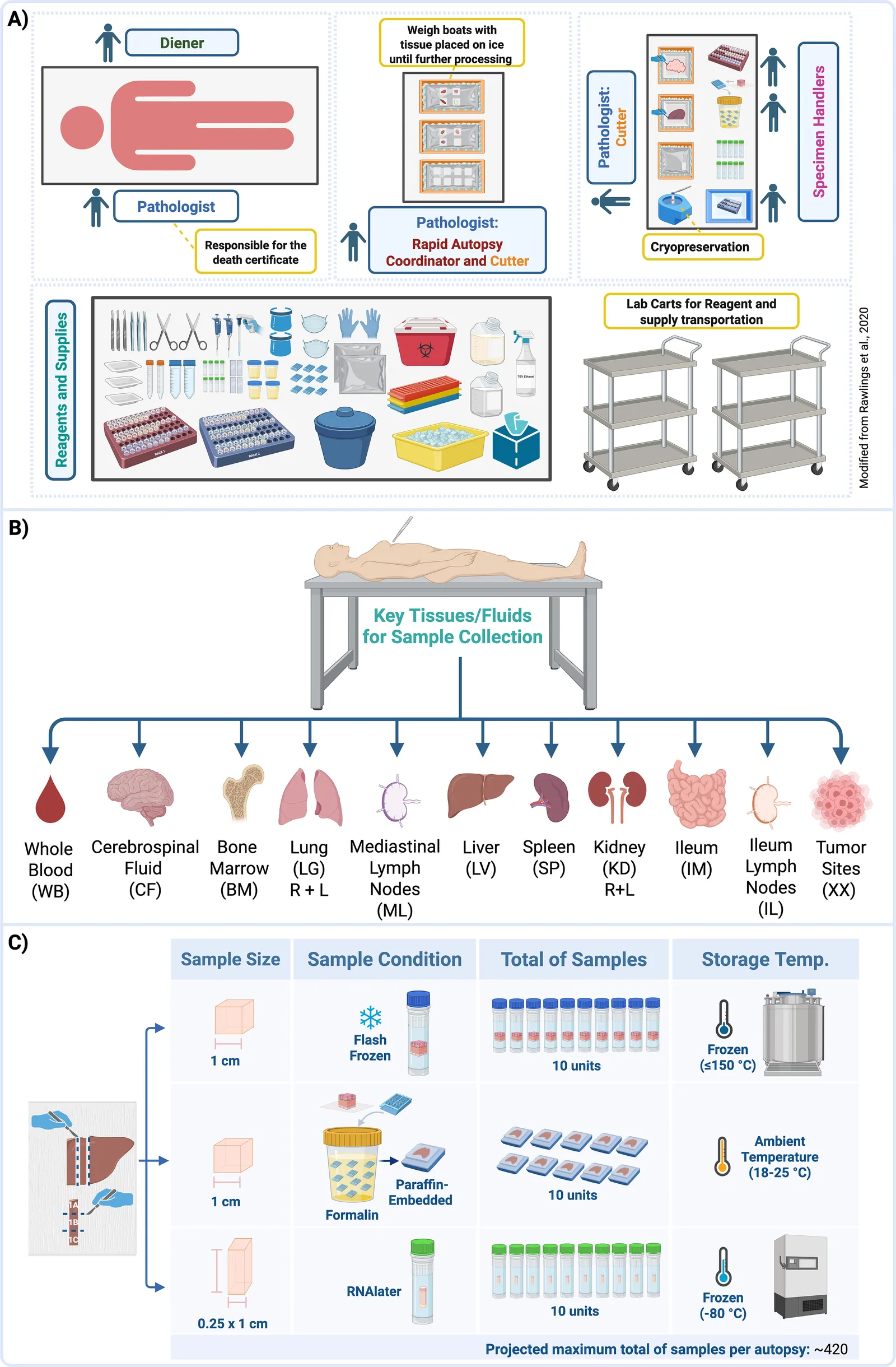
LATAM autopsy program. **(A)** Schematic representation of the autopsy layout. Modified from Rawlings et al.^27^ **(B)** Key tissues/fluids for sample collection. **(C)** Sample size, specimen fixation/preservation methods, expected maximum number of specimens, and storage temperature. LATAM, Latin American.

The study will focus on collecting tissues and body fluids from key anatomical sites, including but not limited to the spleen, cavitary lymph nodes, ileum, heart, kidney, liver, lungs, bone marrow, tumor sites, blood, and cerebrospinal fluid (Fig. 7). For each tissue type, 10 aliquots will be collected, fixed, and stabilized under three conditions: (1) 1 cm^3^ in formalin solution; (2) 0.25 × 1 cm in RNAlater (ThermoFisher Scientific, USA); (3) 1 cm^3^ flash-frozen in LN_2_. Storage will be performed at ambient temperature (18°C–25°C), −80°C after overnight fixation at 2°C–8°C, and at −180°C, respectively. For tumor specimens, sampling will be conducted at both primary and metastatic sites, as well as from uninvolved tissues.

The implementation of this program will significantly strengthen the ACSR’s mission to support HIV-related cancer research. It will offer an unprecedented collection of biospecimens from a racially and ethnically diverse population, including HIV-1 subtypes B and F, which are critical for characterizing viral reservoirs across the human body. By enabling access to tissues otherwise inaccessible via routine clinical biopsies, this initiative will advance the science of HIV/AIDS-associated malignancies and promote equitable, high-impact research.

#### Autopsy program socio-behavioral component

In partnership with the Last Gift Study Team (https://lastgift.ucsd.edu/)—a University of California San Diego research study committed to understanding the behavior of HIV in the human body—the LATAM ACSR has been developing a socio-behavioral initiative titled *A Última Dádiva* (AUD) at the Faculdade de Medicina da Universidade de São Paulo. Through semi-structured interviews, the study aims to explore how socio-behavioral factors influence the acceptability or hesitation toward rapid autopsy for research purposes among PWH with advanced comorbidities receiving palliative care at ICESP. Rapid autopsy is a planned postmortem procedure in which tissue and body fluid samples are collected within a short window after death—typically within 6 hours—to ensure high-quality biospecimens suitable for advanced molecular and cellular analyses. More specifically, the study aims to understand perceptions of rapid autopsy across four key groups: (1) PWH in palliative care; (2) Family members, close relatives, loved ones, friends, or other caregivers; (3) Activists and members of the HIV/AIDS community; and (4) Health care professionals working directly with PWH in palliative care.

AUD builds upon the socio-behavioral framework of the *Last Gift* project. Since its launch in 2017, *Last Gift* has enrolled PWH at the end of life—those with a life expectancy of less than 6 months due to non-AIDS-defining illnesses—as well as individuals with a high estimated 5-year mortality risk but no current terminal condition. To date, approximately 50 participants have enrolled in the Last Gift, many of whom have a history of contributing to HIV clinical research. All participants provided informed consent for tissue and body fluid donation at the time of death via a rapid autopsy protocol, adapted for PWH with terminal illnesses such as cancer, cardiovascular disease, and neurodegenerative disorders. This rapid autopsy approach enables the preservation of high-quality biological materials for studying HIV viral reservoirs and disease mechanisms.^27,33^

In recent years, the *Last Gift* has significantly advanced the literature on the ethical dimensions of end-of-life research with PWH through empirical studies and participant engagement. Five key ethical domains have emerged: (1) protecting autonomy via robust informed consent; (2) avoiding exploitation while promoting altruism; (3) maintaining a favorable benefit-risk balance; (4) safeguarding participants’ vulnerability by centering their values and agency; and (5) fostering acceptance and inclusion of family, loved ones, and community stakeholders.^34^

Within this context, AUD extends the reach of the *Last Gift* by generating culturally and contextually relevant insights into the acceptability of rapid autopsy in Brazil. By engaging diverse perspectives across stakeholder groups, the study aims to inform ethical research practices and policy development for end-of-life studies involving PWH in São Paulo. Ultimately, AUD contributes to advancing ethically grounded, community-informed approaches to HIV research at the end of life. This study was approved on May 26, 2025, by the Research Ethics Committee of the HC-FMUSP, under registration no. CAAE 87546025.5.0000.0068 and ethics approval no. 7.593.022.

### Data and sample management

The ability to effectively manage and integrate specimen data across the ACSR’s RBRs is critical to fulfilling its mission of supporting HIV/AIDS-related cancer research. The ACSR Data Curation & Standardization Pillar (Fig. 2) primary objective is to convert existing RBR inventories into uniform formats, enabling seamless integration into a consolidated, patient-level database. This unified infrastructure forms the backbone of a searchable repository of biospecimens from PWH and cancer. Specimens that have passed quality control and data verification are made available for request through the Letter of Intent (LOI) program.

After completing the patient worksheet, clinical data are collected and maintained using the REDCap (Vanderbilt University, Tennessee, USA) platform, and sample inventory details are entered into Freezerworks—a Laboratory Information Management system. REDCap is a secure, web-based software platform developed to perform data collection and its management. It has tools for data validation, backups, import, and export, while assuring the data quality. Freezerworks is an application used for specimen inventory management that makes it possible to track specimens from collection to distribution, as well as the specimens’ lineage from the original sample to its derivatives.

Both systems require user authentication, and data modifications can be tracked through integrated audit trail functions. The creation of user accounts is restricted to personnel or external guests, conditioned by approval from the institution. Only authorized users can access the databases, ensuring greater security, especially when collecting and exporting data. This dual system facilitates the creation of a centralized biospecimen inventory and a centralized clinical data repository.

Before integration into the centralized repository, three key data verification steps must be completed: (1) physical location confirmation to ensure each specimen includes complete metadata (e.g., freezer, box, and position numbers); (2) informed consent validation to verify that appropriate consent (or documented waiver) exists to permit research use; and (3) distribution status to confirm the specimen is available (i.e., not previously released or depleted). Each RBR site has access to only its data to remain compliant with data use agreements. All clinical details are restricted to defined “allowable” values to promote consistency and enable robust quality control. Upon validation, diagnostic and pathology details provided for each sample are standardized across sites using the 10th revision of the International Classification of Diseases (ICD-10) and the 3rd edition of the International Classification of Diseases for Oncology (ICD-O-3).

The LATAM ACSR also offers a digital library that grants researchers access to a curated collection of digitized pathology images and associated clinical data, supporting remote access and accelerating research.

### Quality management program

The Comprehensive Quality Management Program (QMP) of the LATAM Biorepository is based on a structured, integrated framework designed to ensure the highest operational standards throughout the biospecimen lifecycle—including collection, processing, storage, and distribution. This program is rooted in internationally recognized best practices and guidelines established by the CAP, the NCI Best Practices for Biospecimen Resources, and the ISBER. Key components of the QMP include: (1) SOPs for biospecimen collection, processing, storage, and distribution; (2) annual equipment inspection, qualification, and calibration; (3) continuing education, personnel training, and competency assessments to ensure adherence to current protocols and regulatory requirements; (4) process monitoring and improvement, including periodic reviews of pre-analytical variables and data traceability. The QMP is designed to safeguard specimen integrity, traceability, reproducibility, and compliance with national and international ethical and regulatory frameworks, thus enabling high-quality, reliable research outputs.

### Exploring the ACSR inventory

To support HIV/AIDS-related research, the ACSR offers a structured process for investigators to access high-quality biospecimens as demonstrated by its broad distribution efforts: (1) The ACSR website (https://acsr1.com) allows researchers to explore the current inventory and inquire about specimens before submitting formal requests; (2) the LOI program serves as the gateway for requesting specimens and assessing their availability; and (3) the Letter of Support (LOS) program supports investigators by strengthening grant applications, confirming the availability of biospecimens and associated data.

#### Specimen request and LOI process

Investigators can request specimens for research by visiting the ACSR website (https://acsr1.com). The ACSR has established a LOI program as the formal mechanism for requesting and obtaining samples. For requests involving less than 20 specimens, researchers are directed to submit a small “feasibility” LOI, which requests key study details such as the project title, a brief description, hypothesis, experimental approach, and the type, quantity, and volume of biospecimens requested. Investigators should also specify any relevant study specific criteria (e.g., disease type, treatment history, HIV status). To ensure compliance with ethical and regulatory standards, documentation of Institutional Review Board approval is required for all requests involving clinical samples and de-identified data. A “standard” LOI must be submitted for larger requests involving more than 20 specimens, which receives a higher degree of scrutiny. This includes additional documentation such as a cover letter, a list of potential reviewers, a biosketch, and a statement outlining the significance of the proposed study. To streamline the process, the ACSR also offers an optional pre-submission inquiry. This allows investigators to confirm the availability of specific samples and/or obtain further information about the biorepository inventory before submitting a formal LOI.

#### Back-end process: Exploring inventory and fulfilling requests

Once an LOI is received, the ACSR initiates a structured review and fulfillment process. The standard LOI undergoes rigorous evaluation, including scientific review by the NIH intramural staff of the Office of HIV and AIDS malignancy (OHAM). This review considers several criteria: the types of specimens requested, the appropriateness of proposed techniques, the availability of inventory, and the overall scientific merit of the proposal. This peer-review process is essential for upholding the ethical and scientific integrity of human biospecimens research and conserving specimen inventory. Once the LOI is approved, the ACSR works with the centralized database overseen by the Pillar 2 team (Fig. 2) and the RBRs to identify and select appropriate samples based on inventory availability and LOI parameters. The RBR housing the specimen initiates a Material Transfer Agreement (MTA), and upon receiving a signed MTA from the requesting institution, the ACSR arranges for distribution and covers all associated shipping expenses. In some instances, biospecimens are first concentrated in a single biorepository for processing (e.g., nucleic acid extraction or TMA construction) before final distribution.

#### Letter of support program

Besides providing clinical specimens, the ACSR supports researchers through its LOS program. These letters are offered to investigators submitting grant proposals to major funding agencies, such as the NIH, and serve to confirm the availability of critical biospecimens and de-identified data. By formally demonstrating the feasibility and resource support for a proposed project, LOS letters strengthen applications and underscore ACSR’s commitment to advancing HIV/AIDS research.

#### ACSR distribution output

While utilization rates are commonly used benchmarks in established biobanking sys tems, applying them to a newly launched repository requires caution and consideration of the appropriate context. The LATAM ACSR only began operating at full capacity in December 2023. Since then, efforts have focused on building a robust and well-annotated inventory, establishing sustainable infrastructure, and launching targeted outreach to scientific communities. Given these early stages, crude utilization percentages would not yet provide a meaningful or fair assessment of the repository’s value or trajectory.

**FIG. 8.**
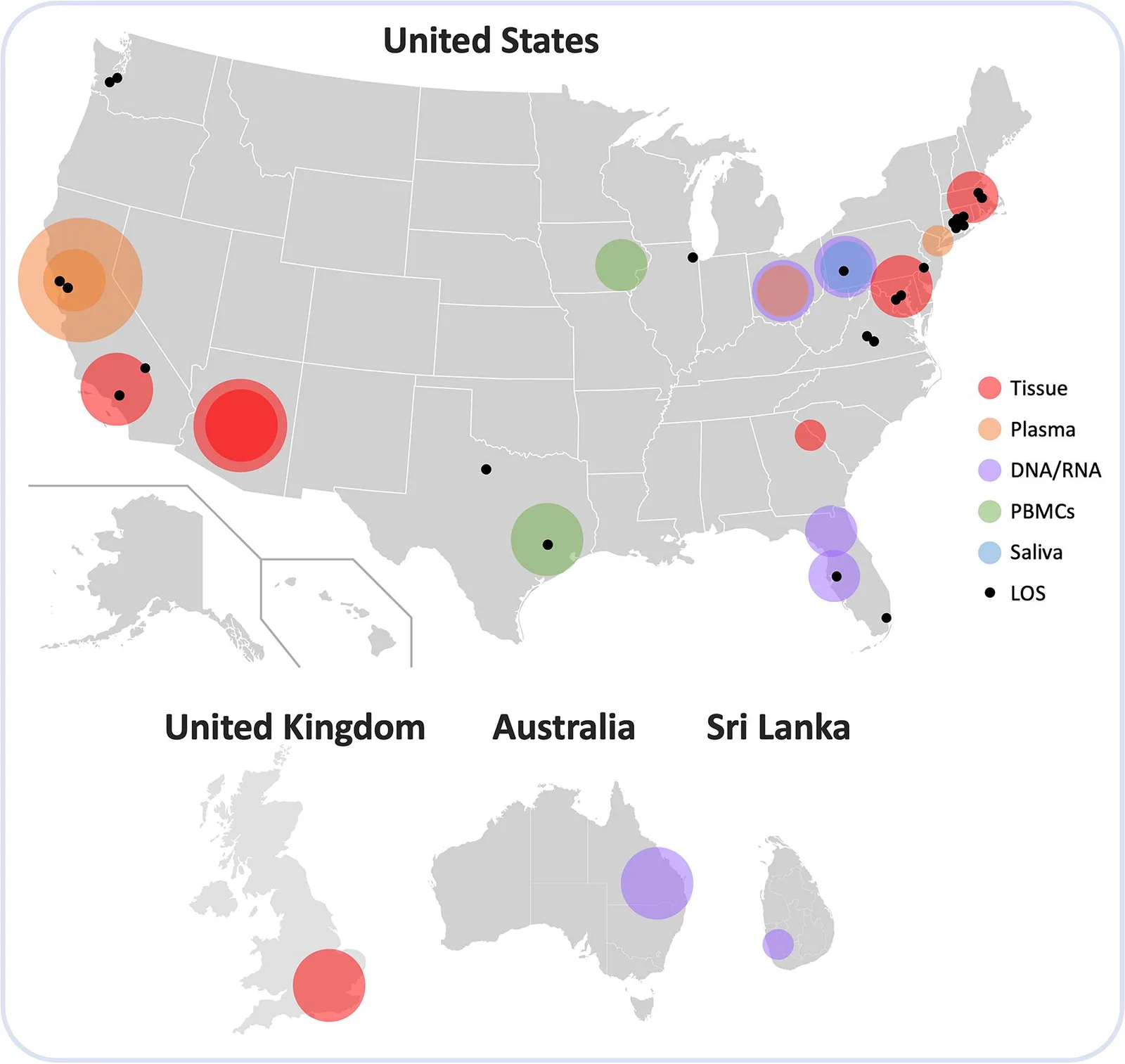
Geographic distribution of ACSR-supported research activities (2022–2024). Map of the United States and international locations illustrating ACSR’s distribution of specimens to various research institutes and universities between 2022 and 2024. The map uses color to denote specimen types—tissue (*red*), blood (orange), DNA/RNA (*purple*), PBMCs (*green*), saliva (*blue*)—while *circle size* reflects distribution volume. Letters of support (*black dots*) are also represented, indicating commitment in providing biospecimen and clinical data in support of grant and/or fellowship applications. ACSR, AIDS and Cancer Specimen Resource; PBMC, peripheral blood mononuclear cell.

In the broader ACSR network, sample utilization is routinely evaluated through downstream impact indicators such as approved requests, contributions to funded research, letters of support, and publications. Between 2022 and 2024, the ACSR distributed over 13,000 specimens of diverse types, including blood, plasma, peripheral blood mononuclear cells, tissue sections, and saliva to national and international research institutions and universities (Fig. 8). It also issued 24 letters of support, with 8 resulting in successful funding. The launch of a unique autopsy program for PWH and cancer is expected to significantly enhance specimen distribution and research capacity across LATAM. This autopsy program is globally unique and has already generated intense scientific interest.

### Concluding remarks

The ACSR is essential for advancing HIV/AIDS research by ensuring equitable access to high-quality biospecimens. Its structured processes and continuous engagement with the scientific community have led to significant research outputs and successful funding outcomes. The ACSR’s commitment to acquiring, storing, and distributing biospecimens for HIV/AIDS research is crucial for advancing scientific understanding of the disease and its related malignancies. The ACSR has developed and implemented standardized protocols and best practices for biospecimen collection, processing, and distribution to uphold scientific integrity and consistency. These protocols integrate quality control measures and utilize state-of-the-art techniques to preserve specimen integrity. Such standardization is vital for reliable results across diverse research efforts. ACSR’s established SOPs are publicly available through the NCI Biospecimen Research Database (https://brd.nci.nih.gov/brd/), providing transparency and valuable guidance to the broader scientific community. ACSR empowers researchers to explore the complex biological and clinical aspects of HIV-associated conditions by ensuring equitable access to high-quality biospecimen resources.

## Authors’ Contributions

L.L.S.S.: Coordinated the LATAM biorepository implementation and oversees its operation; wrote and reviewed the article; prepared figures; is responsible for all communications among authors and with the journal; is the primary author. E.S.M.: Leads surgical pathology and the autopsy program; cowrote the article and prepared figures. K.Y.I.: Coordinates patient enrollment, longitudinal sample collection, and biorepository sustainability; cowrote and reviewed the article. M.U.: Set up state-of-the-art facilities for sample processing, long-term storage and preservation; manages the LATAM biorepository operations; cowrote and reviewed the article. V.M.C.: Pathology department coordinator; supervises sample collection through surgical pathology and autopsies; cowrote and reviewed the article. E.S.M.: biorepository associate; responsible for sample processing, storage, and data management; cowrote the article and prepared figures. C.M.V.M.: Coordinates patient enrollment, longitudinal sample collection, and biorepository sustainability; cowrote the article. G.D.S.: Biorepository nurse; conducts patient enrollment and sample collections; cowrote and reviewed the article. L.O.A.: Biorepository associate; responsible for sample processing, storage, and data management; cowrote the article and prepared figures. I.C.S.: biorepository associate; responsible for sample processing, storage, and data management; cowrote the article and prepared figures. K.O.: Responsible for the LATAM biorepository socio-behavioral component; cowrote and reviewed the article. R.C.: LATAM ACSR coprincipal investigator; oversees the biorepository operations; cowrote and reviewed the article. K.B.: ACSR point of contact for data management and standardization; cowrote and reviewed the article. L.D.: ACSR point of contact for sample distributions; cowrote, reviewed the article, and prepared figures. A.E.C.: ACSR multiple principal investigator; oversees data management and standardization; cowrote and reviewed the article. L.R.: ACSR multiple principal investigator; oversees sample distributions; cowrote and reviewed the article. A.S.: LATAM ACSR principal investigator; leads the biorepository operations; wrote and reviewed the article. J.M.B.: The ACSR principal investigator (contact) obtained funding and directed the implementation of the LATAM biorepository. He is responsible for overseeing all aspects of the biorepository, including financial, ethical, and operational dimensions. He also wrote and reviewed the article.

## Supplemental Material

sj-xls-1-bpb-10.1177_19475535251363861 – Supplemental material for Advancing HIV and Cancer Research: Implementing an AIDS and Cancer Specimen Resource in Latin America Supplemental material, sj-xls-1-bpb-10.1177_19475535251363861 for Advancing HIV and Cancer Research: Implementing an AIDS and Cancer Specimen Resource in Latin America by Larissa L.S. Scholte, Evandro S. Mello, Karim Yaqub Ibrahim, Miyuki Uno, Vanesse Maria da Costa, Ellen Sayuri Miazima, Camila Motta Venchiarutti Moniz, Giullia Dias de Souza, Larissa Oliveira Amorim, Isabela Cristina de Souza, Kris Oliveira, Roger Chammas, Kristina Bowles, Lipsa Das, Anna E. Coghill, Lisa Rimsza, Aluisio Segurado, and Jeffrey M. Bethony
